# Charged Residues in the Membrane Anchor of the Pestiviral E^rns^ Protein Are Important for Processing and Secretion of E^rns^ and Recovery of Infectious Viruses

**DOI:** 10.3390/v13030444

**Published:** 2021-03-10

**Authors:** Kay-Marcus Oetter, Juliane Kühn, Gregor Meyers

**Affiliations:** Institut für Immunologie, Friedrich-Loeffler-Institut, 17493 Greifswald, Germany; K.Oetter@freenet.de (K.-M.O.); kuehnjuliane2609@gmail.com (J.K.)

**Keywords:** pestivirus, RNA virus polyprotein processing, membrane anchor, charge zipper, amphipathic helix, signal peptidase, secreted T2 RNase

## Abstract

The pestivirus envelope protein E^rns^ is anchored in membranes via a long amphipathic helix. Despite the unusual membrane topology of the E^rns^ membrane anchor, it is cleaved from the following glycoprotein E1 by cellular signal peptidase. This was proposed to be enabled by a salt bridge-stabilized hairpin structure (so-called charge zipper) formed by conserved charged residues in the membrane anchor. We show here that the exchange of one or several of these charged residues reduces processing at the E^rns^ carboxy-terminus to a variable extend, but reciprocal mutations restoring the possibility to form salt bridges did not necessarily restore processing efficiency. When introduced into an E^rns^-only expression construct, these mutations enhanced the naturally occurring E^rns^ secretion significantly, but again to varying extents that did not correlate with the number of possible salt bridges. Equivalent effects on both processing and secretion were also observed when the proteins were expressed in avian cells, which points at phylogenetic conservation of the underlying principles. In the viral genome, some of the mutations prevented recovery of infectious viruses or immediately (pseudo)reverted, while others were stable and neutral with regard to virus growth.

## 1. Introduction

Pestiviruses are positive-strand RNA viruses classified as one genus within the family Flaviviridae. The genus Pestivirus contains 11 species (pestivirus A to K) with pestivirus A, B, C, and D representing the long known type species bovine viral diarrhea virus type-1 (BVDV-1) and type 2 (BVDV-2), classical swine fever virus (CSFV), and Border disease virus (BDV), respectively, while the other species comprise more exotic viruses [1,2]. The family also includes the genera Flavivirus, Hepacivirus, and Pegivirus. The pestivirus genome consists of a single-stranded RNA of ~12.3 kb and contains one long open reading frame (ORF) [3]. Translation of the viral genome gives rise to a polyprotein of ~4000 amino acids. Co- and posttranslational processing by cellular and viral proteases leads to 12 mature proteins, arranged in the polyprotein in the order NH2-Npro, C, E^rns^, E1, E2, p7, NS2, NS3, NS4A, NS4B, NS5A, and NS5B-COOH [3]. The pestiviral virion contains the proteins C, E^rns^, E1, and E2 [4,5]. E^rns^, E1, and E2 represent glycosylated envelope proteins exposed on the lipid membrane surrounding the viral capsid. Inside, capsid protein C and the viral genomic RNA are found. All three envelope proteins are essential for generation of infectious virus particles [6,7].

E^rns^ represents a highly glycosylated protein with about 50% of the molecular weight of the mature proteins made up by carbohydrates, which are important for virus virulence [8]. E^rns^ forms disulfide-linked homodimers of ~90 kDa present in infected cells and secreted virions [4,9,10]. Surprisingly, homodimer formation is dispensable for virus viability but has an effect on virus virulence [11,12]. A unique feature of E^rns^ among viral surface proteins is its RNase activity relying on a three-dimensional (3D) structure typical for RNases of the T2 superfamily [6,13,14,15]. Inactivation of the RNase by mutation of active site residues leads to RNase-negative pestiviruses with strongly reduced virulence [16,17]. Since the E^rns^ RNase has been shown to be involved in blocking the host’s type I/III interferon response to pestivirus infection, the attenuation of RNase-negative viruses is regarded as a result of higher susceptibility of the mutant viruses to innate immune mechanisms [18,19,20,21,22,23,24]. This effect of the RNase is especially important for establishment of persistent pestivirus infections [18]. A further unusual feature of this protein is its unusual membrane anchor in the form of a long carboxy-terminal amphipathic helix that aligns in plane with the membrane surface [25,26,27,28]. This kind of membrane association is unknown for cellular surface proteins and has only been reported for one other viral envelope protein so far, the glycoprotein 3 (GP3) of porcine respiratory, and reproductive syndrome virus (PRRSV) [29]. Both E^rns^ and GP3 are secreted to significant amounts by the infected cells [10,25,26,27,29], which, for the pestivirus protein, is believed to be connected with its inhibitory effect on the innate immune system. In infected animals, considerable amounts of E^rns^ are found in the blood [19].

The structural protein region of the polyprotein translated from the pestiviral genomic RNA is processed by cellular signal peptidase (SP) and signal peptide peptidase [10,30]. A typical SP cleavage site is composed of three domains, a usually positively charged N-terminal (n-) region, and a central hydrophobic (h-) region forming an α-helical transmembrane sequence, which is followed by a more hydrophilic part (c-region) containing the actual cleavage site [31,32,33,34]. All of the SP cleavage sites in the pestivirus polyprotein conform to this pattern [3,10,35,36] except for the E^rns^/E1 site, which is lacking a typical h-region, but comprises the carboxy-terminal amphipathic helix of E^rns^ with a terminal von Heijne cleavage motif. This unusual site is nevertheless cleaved by SP, but cleavage is delayed and very sensible to amino acid changes affecting the amphipathic helix [10,26,27,37]. Moreover, the presence of a full-length E1 protein cleaved at its carboxy-terminus by SP is crucial for (efficient) processing at the E^rns^/E1 site [38].

**Figure 1:**
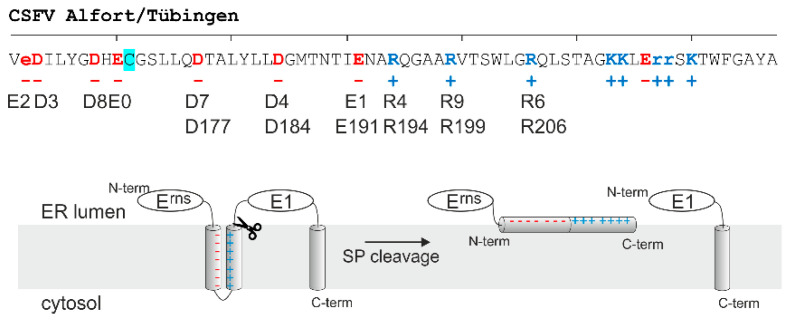
The E^rns^ charge zipper hypothesis. The upper section shows the amino acid sequence of the carboxy-terminal part of E^rns^ form CSFV Alfort/Tübingen. Charged residues are shown in red (negative charge) or blue (positive charge). Cysteine 171, which is responsible for E^rns^ homodimerization, is highlighted with a turquoise background. Above the amino acid sequence, a scale with 10-residue calibration starting with glutamic acid at position 170 is shown. Below the amino acid sequence, the net charges of the residues is given. Below thereof, the designation of the amino acids that were mutated in our studies are given with the short forms used especially in the figures: E2 = Glu162, D3 = Asp163, D8 = Asp168, E0 = Glu170, D7 = Asp177, D4 = Asp184, E1 = Glu191, R4 = Arg194, R9 = Arg199, and R6 = Arg206. For the inner six residues, the full number is also given below. The lower part shows a scheme summarizing the charge zipper theory proposed as involved in processing the E^rns^-E1 precursor. The charge zipper allows the initial formation of a hairpin structure inserted into the membrane, thereby forming a suitable substrate for signal peptidase (SP) cleavage at the E^rns^ carboxy-terminus. After cleavage, the E^rns^ carboxy-terminus is proposed to re-orientate and form an amphipathic helix binding in plane to the membrane surface. The scheme was based on the drawing originally presented in *Cell* by Walther et al. [39], but includes recently published data on the role of E1 for the processing step [38].

The carboxy-terminal sequence of E^rns^ that forms the amphipathic helix contains a set of conserved charged residues (Figure 1). The charges are complementary between the amino- and carboxy-terminal part of the helix so that a hairpin-like structure could establish that brings together the opposite charges. Formation of such a so-called charge zipper was proposed for the E^rns^ membrane anchor in analogy of the bacterial protein TatA, for which charge zippers were described to be responsible for folding and self-assembly of an oligomeric pore structure [39]. In the case of E^rns^, the (intermediate) formation of such a hairpin structure stabilized by up to eight salt bridges is attractive in the context of the carboxy-terminal SP cleavage since the zipped structure of the membrane anchor could re-orientate the helix from the described in plane localization parallel to the membrane surface [25] to a hairpin transmembrane conformation, which would be very similar to the usual SP substrate. We therefore analyzed the influence of mutations affecting the conserved charged amino acids on E^rns^-E1 processing, E^rns^ retention, and release of infectious virus particles.

## 2. Material and Methods

### 2.1. Cells and Viruses

BHK-21 cells (kindly provided by T. Rümenapf) and chicken DF-1 cells (Collection of Cell Lines in Veterinary Medicine (CCLV) of the Friedrich Loeffler Institute) were grown in Dulbecco’s Modified Eagle Medium supplemented with 10% fetal calf serum and nonessential amino acids. Two variants of modified vaccinia virus strain Ankara containing the phage T7 RNA polymerase (MVA-T7), kindly provided by G. Sutter (Ludwig-Maximilians-Universität, München, Germany) and B. Moss (National Institutes of Health, Bethesda, MD, USA) were used in our experiments [40,41].

### 2.2. Construction of Recombinant Plasmids

Restriction and subcloning were done according to standard procedures [42]. Unless stated otherwise, all restriction and modifying enzymes were purchased from New England Biolabs (Frankfurt, Germany) and Thermo Fisher (Karlsruhe, Germany). Synthetic DNA oligonucleotides were synthesized by Metabion (München, Germany).

The plasmids SE^rns^ and SE^rns^-E1 used in expression studies (originally named SSeqE^rns^ and SSeqE^rns^-E1 containing the E^rns^ and E^rns^E1 coding sequences of CSFV Alfort/Tübingen [37,38]) were used as basis for the mutant expression constructs listed in Table 1. Single and multiple point mutations were introduced with standard PCR based methods with thermostable Pfu polymerase (Promega, Heidelberg, Germany) and synthetic primers (QuickChange mutagenesis) in one single reaction or consecutive approaches. The mutated PCR products were all verified by nucleotide sequencing with the BigDye Terminator Cycle Sequencing Kit (PE Applied Biosystems, Weiterstadt, Germany). Sequence analysis and alignments were done with Geneious PrimeR software (Geneious Prime 3 February 2019) (Biomatters, Ltd., Auckland, New Zealand). Further details of the cloning procedures and the sequences of the primers used for cloning and mutagenesis are available on request.

Table 1 lists the mutations tested in the presented work. The first column gives the number specific for each mutation or combination of mutations. The second column indicates the charge pattern present in the encoded proteins. The mirror axis is represented by a slash. The third column shows the amino acid exchanges present in the proteins encoded by the different constructs using the numbering code given in Figure 1. The upper part of the table (A) includes the first four negatively charged amino acids and the respective changes thereof, whereas in (B) only constructs with mutations affecting the inner six charged amino acids are shown. Please note that constructs 10 and 11 are listed in both (A) and (B). In panel (C), mutants are listed in which charged residues were replaced by alanine (a), or a conservative exchange or a different charge exchange were conducted (R4K or R4D, respectively). The given number represents the individual mutation, which can either be present in the E^rns^ expression construct (number followed by “Er”), the E^rns^-E1 expression construct (number followed by ErE1) or the infectious full length cDNA construct (only number).

### 2.3. Transient Expression, Immunoprecipitation, and Quantification of Proteins

BHK-21 cells were infected with vaccinia virus MVA-T7, subsequently transfected with the desired cDNA construct using SuperFect (Qiagen, Hilden, Germany) and labeled with Tran35S-Label (ICN-MP Biochemicals, Eschwege, Germany or Hartmann Analytic, Göttingen, Germany) as described earlier [11]. Supernatant of the cell cultures was harvested for determination of secreted proteins; the cells were washed twice with PBS before cell extracts were prepared under denaturing conditions. Protein expression in equivalent amounts of cell-free supernatant and cell extract was analyzed via immunoprecipitation as described before [27] using monoclonal antibody 24/16 [43] for detection of E^rns^ and the E^rns^-E1 precursor. Precipitates were treated before electrophoresis with 1 µL PNGase F (New England Biolabs, Frankfurt, Germany) for 1 h at 37 °C as suggested by the supplier.

### 2.4. Gel Electrophoresis, Detection, and Quantification of Precipitated Proteins

The precipitated proteins were separated by 10% SDS-PAGE (gel system as published [44]) and E^rns^ or E^rns^-E1 detected and quantified with a Fujifilm BAS-1500 or a CR-35 Bio image plate scanner. The intensities of the signals were determined with TINA 2.0 or AIDA Image Analyser 5 software (equipment and software from Elysia-Raytest, Straubenhardt, Germany). For E^rns^-E1 processing studies, the E^rns^ signal was multiplied with 1.462 to correct for the lower numbers of labeled residues in E^rns^ compared to E^rns^-E1, and the corrected E^rns^ values added to those determined for uncleaved E^rns^-E1 to obtain 100% expression product as a basis for calculation the percentage of precursor. Similarly, the secretion level of E^rns^ expressed from the SE^rns^ constructs was calculated by combining the signals determined for E^rns^ in supernatant and cell extract to obtain 100% expression product for calculation of the percentage of the secreted protein. The presented data represent the averages of at least three independent experiments. Statistical analysis in form of a two-tailed T test was done using the GraphPad Prism software (Statcon GmbH, Witzenhausen, Germany). The same procedure was also used for transient expression of proteins in DF-1 chicken cells, with the exception that Lipofectamine^TM^ 2000 was used for transfection (protocol as recommended by supplier Invitrogen, Karlsruhe, Germany).

### 2.5. Recovery and Analysis of Mutant Viruses from Cloned Sequences

Mutations leading to exchanges of charged amino acids in the E^rns^ membrane anchor were introduced into the CSFV Alfort/Tübingen full-length infectious clone pA/CSFV [45] with standard procedures, as described before [16]. In vitro transcription of RNA from the engineered plasmids and electroporation of cells were done as described before [16]. Replication of RNA and protein expression thereof was detected via immunofluorescence with mab A18 [46] against E2 and FITC-conjugated goat anti-mouse serum (Dianova, Hamburg, Germany). Freeze/thaw extracts were prepared from positive cultures and used for infection of fresh cells. Infection of these cells was detected via immunofluorescence as described above. RNA was isolated from the infected cells with TRIzol^®^ as recommended by the supplier (Invitrogen) and subjected to RT-PCR with primers Ol-E05S (CATGCCATGGGGGCCCTGTTGGCTTGGGCGGTG) and Ol-HPS28.1R (GGACTAGCTTTATAACCTGTCC) using the OneStep RT-PCR kit (Qiagen, Hilden, Germany) and analyzed by nucleotide sequencing as described before [16]. Sequence analysis was done with the Geneious PrimeR software.

## 3. Results

### 3.1. Mutations Interfering with the Putative Charge Zipper Formation Have Inconsistent Effects on E^rns^ Processing

**Figure 2:**
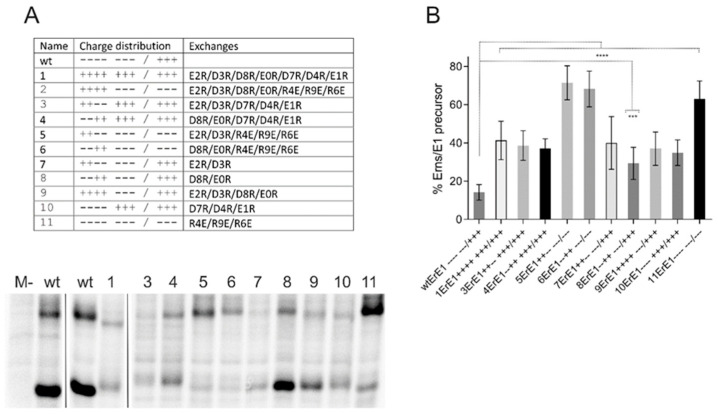
Influence of mutations challenging the E^rns^ charge zipper theory. (**A**) Result of an immunoprecipitation experiment of transiently expressed proteins using E^rns^-specific monoclonal antibody 24/16 with PNGase F treatment of precipitation products prior to separation by PAGE. Characteristics of the expression constructs are given in the table on top, which lists the number of each construct together with the charge distribution pattern and the mutations. The constructs code for: SE^rns^-E1 (wild type E^rns^-E1 fusion protein [38]) or mutants thereof. The code for naming the mutations was already described in Figure 1. The wild type (wt) proteins were loaded twice here because the first two lanes were run on another gel than the other 11 lanes that represent one gel. Original lane with construct 2 was deleted since construct 2ErE1 listed in the table yielded an obviously unstable protein that could not be detected on the gels. M-: mock control (MVA-T7 infection but no plasmid transfection). (**B**) Diagram summarizing the results of immunoprecipitation experiments. The bars represent the percentage of uncleaved E^rns^-E1 precursor determined in at least three independent experiments. For calculation, the radioactivity measured for processed E^rns^ was multiplied with a factor correcting the different numbers of labeled residues in E^rns^ versus the fusion protein E^rns^-E1. This corrected value plus the signal determined for uncleaved E^rns^-E1 was set as 100% recovered expression product, from which the percentage of uncleaved product was calculated. Error bars are indicated and the *p*-value for the results determined for the mutants with respect to the full length E^rns^-E1 is given. **** *p*-value < 0.0001; *** *p* = 0.0004.

Complementarity of the charges left and right of a mirror axis, as found in the membrane anchor sequence of E^rns^ (Figure 1) represents a crucial feature of a charge zipper [39,47]. To test whether the ability to build these salt bridges is important in pestiviruses we established a set of mutants with exchanges that (partially) eliminate complementarity of charges in CSFV expression constructs. In order to put maximal pressure on the system, we replaced charged residues by amino acids with opposite charge, thus, not only preventing the establishment of a salt bridge, but resulting in repulsion. According to the working hypothesis, the charge zipper should be especially important for processing at the E^rns^/E1 site. Since the charge zipper hypothesis is based on the interaction of a whole set of charged amino acids, one would not necessarily expect a dramatic effect of changes affecting individual residues. We therefore started with a variety of mutants with multiple exchanges of charged residues in the amphipathic helix and tested for E^rns^-E1 processing (Figure 2). For the wild type (wt) construct, ~14% of the total E^rns^ protein was found in the unprocessed E^rns^-E1 precursor in steady state. Replacement of all seven negatively charged residues of the inner part of the helix should block any salt bridge stabilized backfolding of the membrane anchor. This mutant showed a significant inhibition of E^rns^-E1 processing (41% of precursor), but the effect was far from a complete block of cleavage as one would expect if backfolding via a charge zipper was a prerequisite for SP cleavage. As expected, less invasive alterations with less than seven exchanges affecting the negatively charged residues resulted in similar, or somewhat reduced, effects on E^rns^-E1 processing. An interesting side effect of these exchanges supporting the strong influence of the charged residues on E^rns^ and E^rns^-E1 structure is the observation of significant alterations in migration behavior of the mutant proteins. Moreover, several mutant constructs yielded significantly less or less stable products (Figure 2). This effect was much less prominent for expression of E^rns^ mutants not containing E1 (compare Figure 2A with Figure 5A), indicating that the mutated precursor E^rns^-E1 might be more readily degraded than the cleaved E^rns^, so that the amount of uncleaved precursor would even be increased for these mutants if degradation was the problem. However, the results were similar after addition of proteasome inhibitor to the cells so that degradation might not be the most important issue.

In addition to exchanges of (part of) the acidic residues, we also tested mutants with alterations affecting the first three positively charged amino acids downstream of the symmetry axis of the proposed charge zipper, either alone or in combination with mutations of acidic residues. Again, all replacements were done with amino acids carrying the opposite charge. Interestingly, all three mutants with negatively charged residues downstream of the symmetry axis exhibited stronger repression of processing than variants with only negative to positive charge changes in the upstream part, regardless of how many of the latter mutations were introduced (Figure 2). The highest effect obtained via changes upstream of the mirror axis was ~41% of uncleaved product, whereas mutations affecting the positively charged residues resulted in a maximum of more than 70% of uncleaved product. These differences were significant with a highest *p*-value of 0.0038 (construct 1ErE1 versus 11ErE1). With regard to the wt protein, all mutants shown in Figure 2 were processed significantly less efficiently with 0.0004, representing the highest *p*-value (construct 8ErE1).

### 3.2. Major Effects Are Observed with Alterations Affecting Only the Inner Six Charged Amino Acids of the Putative Charge Zipper

**Figure 3:**
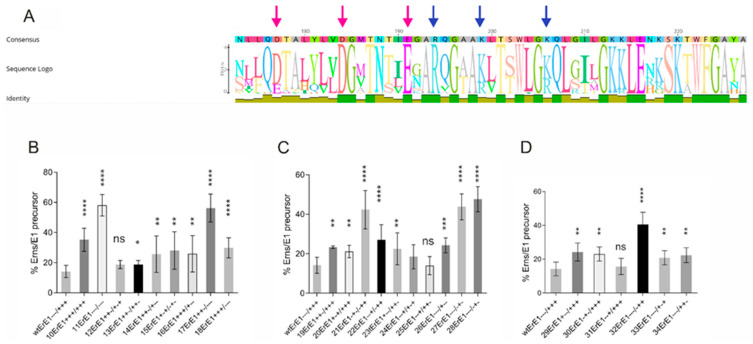
Effect of mutations of the conserved inner six charged residues on E^rns^-E1 processing. (**A**): Graphic representation of the results of an alignment of 67 E^rns^ carboxy-terminal sequences demonstrates the conservation of the inner six charged residues with three negative charges on the left side and three positive charges on the right side of the mirror axis. The size of the letters corresponds to the degree of conservation of the specific residue. The charged amino acids are highlighted by arrows. Please note that the charges are fully conserved even when variation of amino acids is observed. The alignment included BVDV sequences (pestivirus species A and B) as well as CSFV (species C), and Border disease virus (species D). The alignment was established using Geneious Prime software. (**B**–**D**): diagrams summarizing the results of immunoprecipitation experiments after expression of E^rns^-E1 with three or more (**B**), 2 (**C**), or 1 (**D**) changes of the inner six charged residues. The numbers of the constructs together with the charge distribution patterns are indicated at the *X*-axis. The bars represent the percentage of uncleaved E^rns^-E1 precursor determined in at least three independent experiments. The calculation principle is described in the legend to Figure 2, and in Materials and Methods. Error bars are indicated and the *p-*value for the results determined for the mutants with respect to the wt E^rns^-E1 is indicated by asterisks. Significant differences of the E^rns^-E1 processing rate were observed for constructs in (**B**): 10ErE1 and 11ErE1 (*p-*value < 0.0001), 13ErE1 (*p-*value = 0.0271), 14ErE1 (*p-*value = 0.0067), 15ErE1 (*p-*value = 0.0022), 16ErE1 (*p-*value = 0.0062), 17ErE1 (*p-*value < 0.0001) and 18ErE1 (*p-*value < 0.0001). In (**C**): 19ErE1 (*p-*value = 0.0022), 20ErE1 (*p-*value < 0.0019), 21 ErE1 (*p-*value < 0.0001), 22ErE1 (*p-*value < 0.0001), 23ErE1 (*p-*value = 0.0093), 26ErE1 (*p-*value = 0.0006), 27ErE1 (*p-*value < 0.0001) and 28ErE1 (*p-*value < 0.0001). In (**D**): 29ErE1 (*p-*value = 0.0013), 30ErE1 (*p-*value < 0.001), 32ErE1 (*p-*value < 0.0001), 33ErE1 (*p-*value = 0.0081), 34ErE1 (*p-*value = 0.0020). ns = not significant. *p-*value ranges: * <0.05; ** <0.01; *** < 0.001 and **** <0.0001.

Unexpectedly, replacement of (part of) the inner six charged residues of the proposed zipper sequence had very similar effects as changes combining these charges with mutations of further upstream residues (Figure 2: constructs 10ErE1 versus1ErE1, 3ErE1 and 4ErE1 or 11ErE1 versus 5ErE1 and 6ErE1). This could be explained by the zipping process, which should start with residues close to the mirror axis because these residues are located in close vicinity. Thus, stronger effects of exchanges of these residues had to be expected, since residues close to the borders of the helix have a lower probability to get into contact and, thus, should contribute less to establishment of the proposed structure. In accordance with this hypothesis, the charge of these inner six residues is especially conserved among pestiviruses (Figure 3A). We therefore focused on the inner six charged residues flanking the mirror axis for further detailed analysis. Except for mutant D7R/D4R/R9E (construct 12ErE1+ + −/+ − +) all mutants of the initially tested set showed a significantly reduced E^rns^-E1 cleavage efficiency (Figure 3). When three or more of the six inner charged amino acids were replaced by residues with the opposite charge the amount of uncleaved E^rns^-E1 precursor ranged from wt level (~15%, construct 12ErE1+ + −/+ − +) to ~58% (construct 11ErE1 − − −/− − − with mutations R4E/R9E/R6E). Regarding the hypothetical charge zipper to be formed by these residues, the position of the exchanges does of course matter. Therefore, the different mutants had to be evaluated with regard to their ability to establish salt bridges to stabilize the predicted hairpin structure. Three of the constructs preserved the possibility that the inner six charged residues form three salt bridges because of reciprocal exchanges (14ErE1+ + −/+ − −, 15ErE1+ − +/− + − and 18ErE1+ + +/− − − (Figure 3A)). These constructs show a moderately but significantly reduced E^rns^-E1 cleavage efficiency. Four of the other mutants were able to form two salt bridges (12ErE1+ + −/+ − +, 13ErE1+ + −/+ + −, 16ErE1+ + +/+ − − and 17ErE1+ + −/− − −). Among these, three mutants showed again only slightly but significantly increased amounts of uncleaved E^rns^-E1. In contrast, cleavage efficiency of the variant 17ErE1+ + −/− − − was dramatically reduced (56% of uncleaved precursor versus 14% for the wt). Surprisingly, the two already presented constructs with exchanges preventing all three ionic interactions showed significantly differing results with the all-positive version 10ErE1+ + +/+ + + being much less affected than the all-negative mutant 11ErE1− − −/− − − (35% versus 58% with a *p*-value < 0.001).

The above-described results revealed a variable effect of the different mutations, which were not fully congruent with the charge zipper hypothesis. For further elucidation, we tested also mutants with a lower number of exchanges. Ten versions with two mutations and six with a single change were included (Figure 3C,D). The effects of two exchanges were quite similar to those determined for mutants with three or more changes. Except for 24ErE1− − +/+ − + and 25ErE1− − +/+ + − all mutants resulted in significantly reduced E^rns^-E1 cleavage efficiency. The amount of uncleaved precursor determined for the two residue mutants varied between wt level (about 14% uncleaved product) and about 50% precursor, and thus showed somewhat reduced maximal values compared to the mutants with multiple exchanges. Again, a significant reduction of processing activity was also observed for a mutant preserving the ability to form all proposed salt bridges (22ErE1− − +/− + +). Constructs 21ErE1− + −/− + +, 27ErE1− − −/− − + and 28ErE1− − −/− + − exhibited especially strong reduction of cleavage efficiency almost reaching the levels of the two highest affected mutants of the set with more than two alterations, 11ErE1− − −/− − − and 17ErE1+ + −/− − −.

Except for 31ErE1− − +/+ + + also the single exchanges resulted in significantly reduced E^rns^-E1 processing (Figure 3D). Again, the level of reduction was somewhat lower than for the variants with two or more changes, but the highest amount of E^rns^-E1 precursor observed for the 32ErE1− − −/− + + mutant was still above 40% of the total protein compared to 14% of the wt. Taken together, most of the tested charge exchanges reduced the E^rns^-E1 processing rate, but the number of possible salt bridges in the mutant sequences did not correlate with the degree of impairment of cleavage efficiency.

### 3.3. Effects of Less Invasive Alterations

Most of the mutations described above had significant influence on E^rns^-E1 processing, but it has to be kept in mind that the introduced changes with the opposite charge were fundamental. The least invasive alteration that could be introduced into the amphipathic helix would be the replacement of charged residues by different amino acids with the same charge (e.g., exchange K for R or E for D). If charge mattered, these changes should be neutral, whereas the exchange of any negatively charged amino acid for any positively charged residue or vice versa should lead to an equivalent impairment of processing. We, therefore, tested a selected group of mutants reflecting the above-described two groups with preservation of the same charge or replacement by both possible residues with opposite charges. Constructs with charge preservation exchanges behaved similar to the wt, which result could be expected, since such exchanges also occur naturally in different virus strains (see Figure 3A). In contrast, introduction of the opposite charge had principally the same effect, regardless, which of the two possible amino acids were chosen (data not shown). Thus, it can be concluded that the effects of the mutations on processing resulted primarily from a change of charge and not from replacement of a specific amino acid.

**Figure 4:**
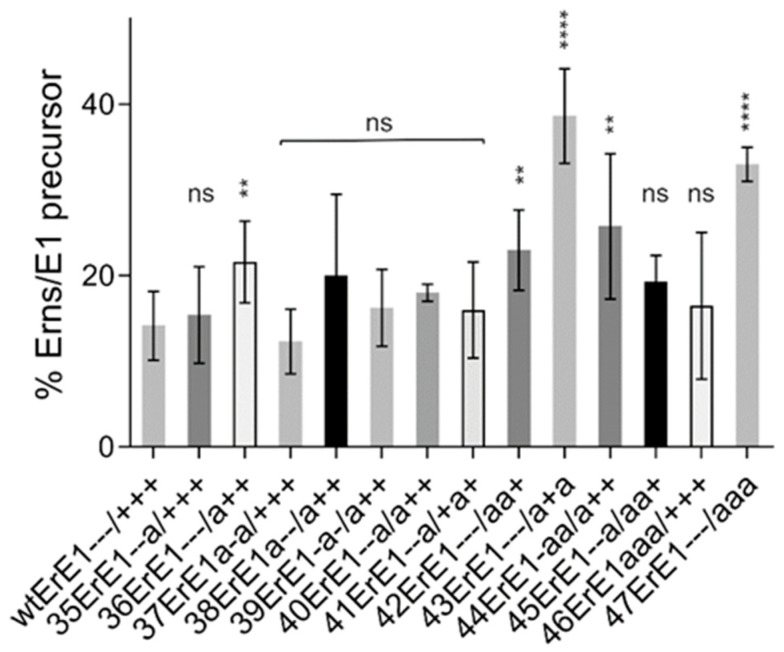
Replacement of charged residues by alanine. Diagram summarizing the results of immunoprecipitation experiments. The bars represent the percentage of uncleaved E^rns^-E1 precursor determined in at least three independent experiments and calculated as described above. Error bars are indicated. Constructs with significantly different processing efficiency compared to the wt: 36ErE1 and 44ErE1 with *p-*value of 0.0014, *p* = 0.0026 for 42ErE1− − −/aa +, and *p* < 0.0001 for constructs 43ErE1− − −/a + a and 47ErE1− − −/aaa. ns = not significant. *p-*value ranges: ** <0.01, and **** <0.0001.

The type of the tested exchanges with introduction of the opposite charge of the wt was selected because we wanted to challenge the charge zipper model by destroying possible salt bridges or support it by double exchanges, preserving the ability to form such bridges. Since the results did not support this idea, we wondered whether other exchanges might have an effect. It could be that not the nature of the charge alone, but also the presence of polar hydrophilic residues at the respective positions was the main important point. We therefore established some constructs, in which charged residues were replaced by the neutral residue alanine. In all cases, the alanine mutations had less prominent effects on E^rns^-E1 processing than the corresponding charge exchanges (Figure 4). The majority of the constructs showed no or rather moderate changes compared to the wt with the strongest effects detected for some constructs with more than one mutation. Since the replacement of charged residues by alanine, though less invasive than introduction of a residue with opposite charge, nevertheless prevents the establishment of salt bridges, these results again support the conclusion that salt bridges between complementary charged amino acids in the E^rns^ membrane anchor cannot be of major importance for E^rns^-E1 processing.

### 3.4. Exchange of Charged Residues in Its Membrane Anchor Can Lead to Increased E^rns^ Secretion

**Figure 5:**
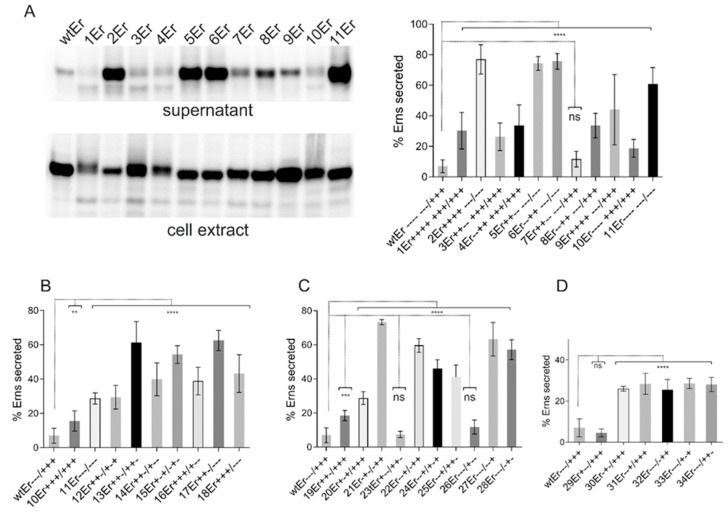
Exchange of charged residues in the membrane anchor increases E^rns^ secretion. Results of immunoprecipitation experiments from supernatant or extracts of cells transiently expressing E^rns^ (from construct SE^rns^ [38]) or mutants thereof with exchanges affecting the charged residues in the E^rns^ carboxy-terminus using E^rns^ specific mab 24/16. In (**A**), a representative gel with PNGase F treated proteins precipitated from the supernatant and cell extract is shown on the left, whereas the diagram on the right summarizes the results of at least three independent experiments quantified via phosphorimager analysis. The number of the individual construct together with the charge distribution in the E^rns^ carboxy-terminus are given at the *X*-axis (see also Table 1 for information concerning the mutations). The bars represent the amount of secreted protein as percent of total recovered expression product. For calculation, the counts determined for extra- and intracellular E^rns^ were set to 100% expression product as basis for calculation of the secretion value. Error bars are indicated as well as the *p*-value of E^rns^ mutants compared to E^rns^ wt with **** representing *p* < 0.0001. (**B**–**D**) summarize the results of immunoprecipitation experiments after expression of E^rns^ with three or more (**B**), 2 (**C**) or 1 (**D**) changes of the inner 6 charged residues. The numbers of the constructs together with the charge distribution patterns are indicated at the *X*-axis. For all tested constructs with exchanges of three or more of the inner six charged amino acids (**B**) significant differences of the E^rns^ secretion rate, with regard to the wt control, were observed with *p-*values of 0.002 for 10Er and *p* < 0.0001 for the other constructs. In case of two exchanges (**C**), two constructs (23Er and 26Er) show no significant difference to wt, whereas the *p-*value for 19Er was 0.0003 and <0.0001 for the other constructs. For constructs with one exchange (**D**) only 29Er did not yield a significant difference to wt. All other constructs showed values of *p* < 0.0001. ns = not significant. *p-*value ranges: ** <0.01; *** <0.001.

The amphipathic helix of E^rns^ is not only important for processing at the E^rns^/E1 site but is also crucial for E^rns^ membrane anchoring and intracellular retention of the protein [3,25,27,28,37,48]. Mutations disturbing the amphipathic character of the carboxy-terminal helix lead to significantly increased secretion of E^rns^ [27]. So far, the importance of the conserved charged residues in the amphipathic helix for the retention/secretion equilibrium is not known. Therefore, we introduced a selected set of the above-described mutations into an E^rns^ expression construct and compared the secretion level with that of wt E^rns^. We started with mutants equivalent to those analyzed in Figure 2 with up to seven exchanges in the set of the seven negatively charged residues upstream of the mirror axis and the three arginines downstream thereof. Except for mutant 7Er with exchanges affecting the first two negatively-charged residues, all of the tested mutants showed significantly increased E^rns^ secretion, even though the extent of this increase was somewhat variable (Figure 5A). As already observed when analyzing E^rns^-E1 processing, exchanges affecting only the inner six residues had similar effects as mutations including replacement of the four upstream negatively charged amino acids. We, therefore, again concentrated on the inner six charged residues during further analysis. Except for the mutation D177R (mutant 29E+ − −/+ + +), all exchanges of single amino acids with opposite charge had significant effects on E^rns^ secretion with at least doubling the amount of extracellular E^rns^ (Figure 5B). Replacement of up to six of these residues by amino acids with opposite charge had variable effects with some mutants exhibiting about wt level or moderately increased secretion rates, whereas strong increases up to ~75% of E^rns^ in the supernatant were detected for others (Figure 5B–D). An interesting observation already made when analyzing E^rns^-E1 processing was the in general higher effect of mutations affecting the arginines downstream of the mirror axis. Apart from the frequent presence of the R194E mutation in highly secreted mutants (see below), a common pattern correlated with high secretion levels was not obvious. As also seen for the processing step, a general tendency towards higher secretion rates was observed when more than one charge was changed, but the introduction of more than two alterations did not automatically lead to higher secretion rates than two changes.

The most drastic changes affecting the inner six charges with all positive, all negative, or full reversion of these charges, did not reduce the amount of intracellular E^rns^ stronger than some single exchanges. It, therefore, can be concluded that the conserved charged amino acids in the E^rns^ membrane anchor are important for E^rns^ membrane binding and retention, but the ability to establish a hairpin structure stabilized by salt bridges is not relevant for the equilibrium of E^rns^ retention/secretion.

**Figure 6:**
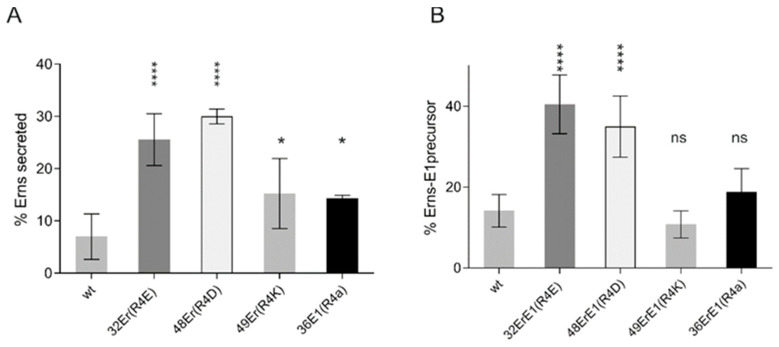
Arg194 alone has a significant impact for E^rns^ secretion and E^rns^-E1 processing. Summary of the results of immunoprecipitation experiments with supernatant and extract of cells transiently expressing E^rns^ or E^rns^-E1 or mutants thereof affecting Arg194. The numbers and mutations of the expressed constructs are given at the *X*-axis. The bars show the mean values of phosphorimager analyses based on at least three independent experiments for E^rns^ secretion (**A**) and E^rns^-E1 processing (**B**) with standard deviation. Significant differences with regard to the wild type are indicated with asterisks. Er36 * *p* = 0.014, Er43 **** *p* = 0.0119. ns = not significant.

Comparison of all the results obtained for different mutants highlighted that a surprisingly high percentage of the constructs showing above average effects contained a replacement of arginine 194. Figure 6 summarizes the results of different single exchanges at this position. Both retention of E^rns^ and E^rns^-E1 processing were significantly reduced when only this residue was replaced by an acidic amino acid, whereas exchanges for lysine or alanine resulted in only a slight significant increase of E^rns^ secretion and had no significant effect on E^rns^-E1 processing, which reflects the basic conclusions of the mutagenesis analysis that the presence of the correct charge is the important feature.

### 3.5. The Principles of E^rns^-E1 Processing and Secretion Are Conserved between Mammalian and Avian Cells

**Figure 7:**
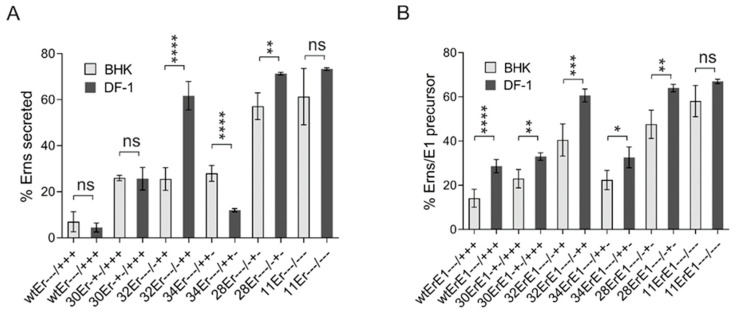
Importance of charged amino acids for E^rns^ retention and E^rns^-E1 processing in avian cells. (**A**) Comparison of the results of immunoprecipitation experiments from supernatant or extracts of mammalian BHK-21 with chicken DF-1 cells transiently expressing E^rns^ (wtEr, construct SE^rns^ [38]) or mutants thereof with exchanges affecting the charged residues in the E^rns^ carboxy-terminus using E^rns^ specific mab 24/16. The diagram summarizes the results of at least three independent experiments quantified via phosphorimager analysis. The number of the individual construct together with the charge distribution in the E^rns^ carboxy-terminus are given at the *X*-axis (see also Table 1 for information concerning the mutations). The bars represent the amount of secreted proteins as percent of total recovered expression product calculated as described above. Error bars are indicated as well as the *p*-value of the mammalian compared to avian cells with **** representing *p* < 0.0001 whereas ns means not significant. *p*-value for 28Er: 0.0046. (**B**) Same as in (**A**), but determination of E^rns^-E1 processing efficiency after expression of constructs ErE1 (based on plasmid SE^rns^E1 [38]). The bars represent the percentage of uncleaved E^rns^-E1 precursor determined in at least three independent experiments and calculated as described above. Error bars are indicated and the *p*-value for the results determined for the mammalian versus avian cells for each construct is given. For all tested constructs, significant differences between cell lines were observed except for plasmid 11ErE1. *p*-values: 30ErE1: 0.0085; 32ErE1: 0.0003; 34ErE1: 0.02; 28ErE1:0.0022. Please note that constructs 30ErE1 and 34ErE1 do not show significant differences to construct wtErE1 in the mammalian cells in contrast to avian cells. *p*-value ranges: * <0.05; ** <0.01; *** <0.001 and **** <0.0001. ns = not significant.

Signal sequences and their processing by SP were highly conserved during evolution, so that mammalian signal sequences are usually functional also in, e.g., avian or insect cells. Since, however, the E^rns^ membrane anchor represents a highly unusual substrate for SP additional factors could be necessary for processing of E^rns^-E1, which might not be evolutionary conserved. Similarly, also establishment of E^rns^ membrane anchoring and intracellular retention might be dependent on specific host factors, which could rely on the conserved charged residues. We were, therefore, interested to analyze E^rns^-E1 processing and E^rns^ retention as well as the effects of charge mutations in evolutionary distant cells from a species not known as host of pestiviruses and, therefore, conducted experiments in avian cells. Since avian cells can be infected with Vaccinia virus MVA, we could use the same plasmids as for the other experiments and induce T7 RNA polymerase driven expression via MVA-T7. A selection of constructs with single, double, or triple exchanges were tested in the chicken cell line DF-1. Expression of E^rns^ wt and mutants allowed to determine the secretion rate, which was affected by the tested changes very similarly in avian and in BHK-21 cells (Figure 7A). In fact, most of the results were strikingly similar in both cell types. Only the two single Arg exchanges showed different levels of secretion increase with a much higher influence of R194E and a significantly lower influence of R206E in avian cells compared to the BHK-21 cells. However, in both cases, the general effect, namely the strongly increased secretion rate, was obvious for both mammalian and avian cells. There is no clear tendency detectable when E^rns^ secretion in BHK-21 and DF-1 results are compared overall, because in a given cell line some mutations show increased secretion levels while others were less prominent in the supernatant. When the processing of E^rns^-E1 was analyzed, consistently less efficient cleavage was observed in the avian compared to the mammalian cells (Figure 7B). The difference is in all cases between ~10 and 20% reduction of the processing rate in the avian cells, and is significant for all tested constructs except for 11ErE1. Importantly, the tendency of the influence of the different mutations is the same in both cell types. Taken together, also in the phylogenetically distant avian cells, replacement of charged residues in the E^rns^ membrane anchor by amino acids with opposite charge led to increased levels of E^rns^ secretion and reduced rates of E^rns^-E1 processing indicating that the underlying principles are conserved.

### 3.6. Effects on the Recovery and Viability of Infectious Viruses

**Figure 8:**
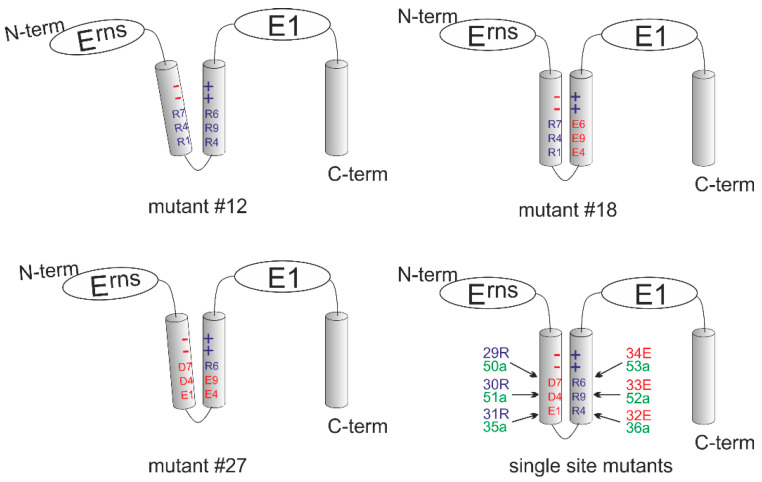
Schematic presentation of the mutants included in Table 2. The four schemes show the putative charge zipper region of E^rns^ for the mutants of Table 2 with multiple exchanges (#12: upper left, #18: upper right; #27: lower left) and a summary of all single mutations (lower right). Amino acids are as described above (D7 = Asp177, D4 = Asp184, E1 = Glu191, R4 = Arg194, R9 = Arg199, and R6 = Arg206). Red color symbolizes negative charge, blue color positive charge and green no charge. The summary of the single exchange mutants shows the wt configuration of the proposed charge zipper with the number of the mutants and the respective amino acid on the left or the right. The tilted helices shown for mutants #12 and #27 indicate the different degree of repulsion induced by the charge changes.

**Figure 9:**
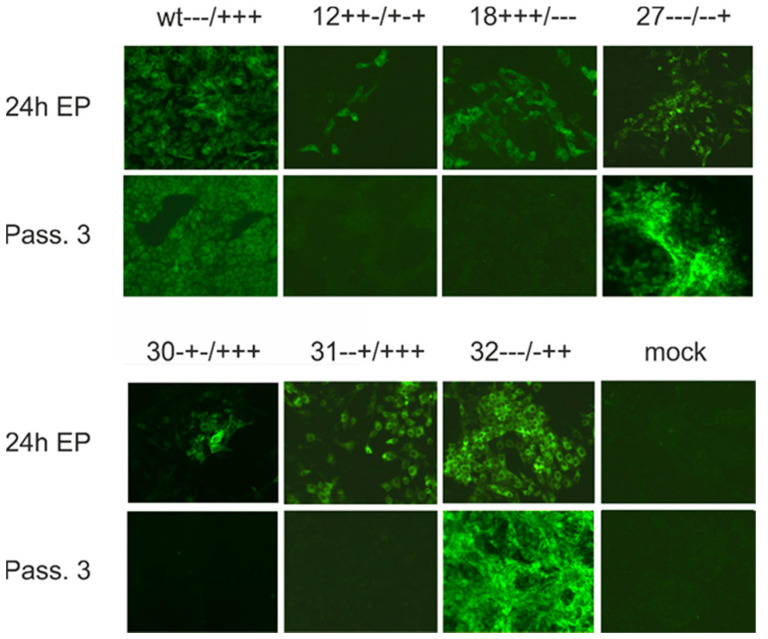
Exchange of charged residues in the E^rns^ membrane anchor can block production of infectious virus. Immunofluorescence analyses conducted after transfection of CSFV genome-like wt RNA or RNA with the given selected exchanges of codons for charged amino acids in the carboxy-terminal region of E^rns^. The upper panel shows mutants with more than one exchange and the panel below shows single exchange mutants. In each panel, the upper row shows results of analyses conducted ca. 24 h post electroporation and proves that all tested RNAs represent functional replicons. The lower row shows the results obtained after three passages of cell extract used for infection of fresh cells. Immunofluorescence was done with mab a18 directed against the E2 protein. Please refer to Table 1 for decoding the analyzed mutations.

Taken together, our mutagenesis analyses prove that the conserved charged residues in the E^rns^ membrane anchor have an influence on E^rns^-E1 processing and intracellular retention of E^rns^, but are not of crucial importance per se. Loss of single charged amino acids has only minor or moderate effects and even mutation of several charged residues does not block E^rns^-E1 processing completely. The maximal effect on E^rns^ secretion rate in consequence of multiple exchanges was in the same range as observed for insertions impairing the amphipathic character of the E^rns^ carboxy-terminus [27] or carboxy-terminal deletions of the E1 moiety in the E^rns^-E1 precursor [38]. The general level of the observed effects on processing and retention, and especially its dependence on the position of the alteration in the amphipathic helix do not support an obvious general importance of the conserved charge distribution. We, therefore, introduced a selected set of our mutations into the full-length cDNA copy of the CSFV Alfort/Tübingen genome [45], and tried to recover infectious viruses upon transfection of in vitro transcribed genome-like RNA. We selected mutants with moderate effect on secretion, and only low change of processing rate (e.g., mutant 18+ + +/− − − with exchanges D7R/D4R/E1R/R4E/R9E/R6E, mutant 12+ + −/+ − + with exchanges D7R/D4R/R9E and construct 31− − +/+ + + with E1R), moderate influence on both secretion and processing (e.g., mutant 32− − −/ − + + = R4E) and strong alteration of both parameters (e.g., mutant 27− − −/− − + with R4E/R9E). In addition, we also tested some single alanine mutations that had mostly shown no significant influence on E^rns^-E1 processing. Table 2 presents the complete list of the tested constructs and Figure 8 shows the influence of the changes with regard to the putative charge zipper. Transfection of RNA derived from the full-length plasmids resulted in detection of viral protein in the transfected cells when tested ca. 24 h post electroporation (representative examples shown in Figure 9). This finding proved replication of the transfected RNA since protein translated from input RNA is not detectable in our system as shown before [45]. Thus, the respective RNAs were at least functional as replicons. As a next step, freeze/thaw extracts of the transfected cells were generated and used for infection of fresh cells. This step was repeated three times and after three passages, the cells were tested by indirect immunofluorescence for expression of viral proteins. Positive results were obtained for the RNAs with the wt sequence and the mutants with only single exchanges except for mutants 30− + −/+ + + (D4R) and 31− − +/+ + + (E1R). Among mutants with more than one replaced amino acids only the double mutant 27− − −/− − + (R4E/R9E) allowed recovery of infectious viruses, whereas the other mutants and the mock control were negative (Table 2).

To test whether the introduced alterations were stable, we isolated RNA from cells of the third passage infection with these viruses. RT-PCR and nucleotide sequencing revealed reversions or pseudoreversions for mutants 27− − −/− − + (R4E/R9E), 32− − −/− + + (R4E), 34 − −/+ + − (R6E), and the Ala mutants 35 − − a/+ + +, 36 − − −/a + + and 51-a −/+ + + (with E1a, R4a and D4a, respectively) (Table 2). These analyses clearly showed that even single charge exchanges can block production of infectious pestiviruses. The pseudoreversions stress the importance of a positive charge at the respective positions showing that the correct charge is more important for virus replication than the specific amino acid. To analyze the replication characteristics of mutant viruses with stable exchanges, we recorded growth curves of mutants 29 + − −/+ + + (D7R), 33 − − −/− + − (R9E), and 53 − − −/− − a (R6a) in comparison to the wt virus. These three mutants showed wt growth characteristics, which result further supports that these alterations do not hamper the virus and, thus, are stable.

## 4. Discussion

The E^rns^ protein of pestiviruses is a unique achievement of viral evolution that combines functions of an essential structural protein with an enzymatic activity involved in controlling the host’s innate immune response to pestivirus infection. The E^rns^ RNase activity represents an important virulence marker of pestiviruses and is one component of a complex strategy resulting in establishment of persistent infection as best studied for BVDV [3,16,17,18]. Part of the E^rns^ protein synthesized within cells is secreted to the cell-free supernatant and this feature is hypothesized to be connected with its function as an inhibitor of the type-1 interferon (IFN-1) response together with its RNase activity [10,19,20,22,24]. E^rns^ is bound to membranes via an unusual membrane anchor composed of a long amphipathic helix anchor located at its carboxy-terminus. This helix associates in plane with the lipid bilayer surface [25,26,27]. Among surface proteins, this type of membrane anchor is only found in E^rns^ and the GP3 envelope protein of PRRSV [29]. Association to membranes via the amphipathic helix is believed to be responsible for partial secretion of both of these proteins, ensuring an equilibrium between secretion and retention. This equilibrium is necessary to release a significant amount of these proteins into the supernatant and at the same time retain enough of these structural components for virion formation at the site of virus budding within the cell. In pestiviruses, the secreted protein helps blocking the IFN-1 response to virus infection, which is supported by tissue culture and in vivo experiments although the underlying mechanisms are not yet fully understood [18,19,20,22].

In addition to the establishment of an equilibrium between E^rns^ retention and release the amphipathic helix has also to support processing at the E^rns^/E1 site since the gene expression strategy of pestiviruses relies on translation of a polyprotein from a single ORF and subsequent proteolytic cleavage. As true for a variety of enveloped positive-strand RNA viruses, pestiviruses employ cellular SP and SPP for processing of their structural proteins [10,30,37]. Since the in-plane orientation of the amphipathic helix stands in marked contrast to the standard arrangement of SP cleavage substrates [31,32,33,34], the E^rns^/E1 site represents a highly unusual SP cleavage site. We have shown before that the integrity of the amphipathic helix as well as the presence of a full length E1 are crucial for efficient E^rns^-E1 processing [37,38]. The finding of a conserved set of complementarily charged amino acids in the amphipathic helix able to form a so-called charge zipper seemed to shed some light on the unusual processing mechanism. As proposed, the charge zipper could stabilize a hairpin structure with hydrophobic outer surfaces so that it could insert into a membrane spanning the lipid bilayer twice [39]. In this configuration the sequence directly upstream of the E^rns^/E1 cleavage site would adopt a structure somewhat reminiscent of a standard SP cleavage site and could thus promote or at least facilitate cleavage by the cellular protease.

Based on this hypothesis, we analyzed the effect of replacing charged residues by amino acids with opposite charge, the same charge, or neutral without charge (alanine) on processing of the E^rns^-E1 precursor. Exchanges preserving the charge and most alanine substitutions had no (or only very small) effects on processing efficiency, whereas some mutants containing replacements with opposite charges were severely impaired. Nevertheless, the results contradict the charge zipper hypothesis since the outcome of mutations was dependent on the positions of the exchanges with R194E representing the most effective change. In general, replacement of the positively charged amino acids by acidic amino acids resulted in stronger effects than mutations affecting the negatively charged residues. This is especially obvious for the construct with the three inner arginines replaced by glutamic acid compared with the reciprocal mutation (inner three acidic residues replaced by arginine). From a charge zipper aspect both should be equal, but the reduction of cleavage seen for the latter was much lower. Similarly, the highest amount of unprocessed precursor seen for a two residue change (R4E/R6E) was ca. 50% of the total protein, whereas double mutants D7R/D4R or R9E/R6E are much less affected, even though all three mutants can establish only one out of three inner salt bridges. Moreover, we did not see a general additive effect when the number of destroyed salt bridges increased. The mutants E1R/R9E and E1R/R6E with only one inner salt bridge display about wt processing levels whereas D7R/D4R/R4E/R9E/R6E exhibits ca. 50% reduced level although it can form two. There are a lot more examples where the determined E^rns^-E1 cleavage efficiency differs from the effects expected in consideration of the charge zipper hypothesis.

Similar results as described for the processing of the E^rns^-E1 precursor were also obtained when the influence of charge exchange mutations in the E^rns^ membrane anchor on secretion of E^rns^ was analyzed. While the E^rns^ membrane anchor was shown to be bound in plane with the membrane surface [25], one could hypothesize that a transient hairpin structure with a hydrophobic outer surface formed via a charge zipper could represent an intermediate during establishment of the membrane contact. However, the intensity of E^rns^ secretion did not correlate with the number of salt bridges destroyed by the mutations. In general, the observed changes were somewhat more prominent than those observed for the processing step, a fact that is more obvious for single and double mutants than for variants with multiple exchanges. The stronger influence of the mutations on secretion might reflect a kinetic effect. As we have shown before, the E^rns^-E1 precursor is bound to the ER membrane via the transmembrane structure at the carboxy-terminus of E1 [38]. Thus, processing can be achieved over a longer time period so that also suboptimal sites could be cleaved with rather good activity. In contrast, an E^rns^ anchor mutant with reduced membrane affinity could be imagined to leave the ER on the secretory pathway and, thus, be bound for secretion, rather quickly leading to kind of an “all or nothing process”.

In light of the unusual features of the E^rns^ carboxy-terminus, both with regard to membrane anchor function and substrate for SP cleavage, it was quite surprising that these features are also working in phylogenetically clearly distinct avian cells, which are no natural host for pestiviruses. This finding shows that the E^rns^ membrane anchor relies on evolutionary conserved mechanisms and does not employ factors that are specific for e.g., mammals. This becomes even clearer, when considering that the effects of mutations affecting the charged residues are also very much comparable between both species. It has already been shown before that SP cleavage is conserved between mammals and birds and important analyses of SP have been conducted with enzyme isolated from avian cells [49,50,51,52]. However, the E^rns^/E1 cleavage site represents a really special substrate so that involvement of specific factors for its processing would not have been a big surprise.

As outlined above, neither the E^rns^-E1 precursor processing nor the E^rns^ membrane binding seem to be dependent on the charged residues in the amphipathic helix in a way that would strongly support the charged zipper hypothesis. Conservation of the charges could in part, of course, be due to overlapping effects, when, e.g., a charged residue is not only important for the zipper but has also other functions. The basic amino acids found in quite high frequency in the carboxy-terminal part of the helix can interact with the negatively charged head groups of the phospholipids and, therefore, be more important for the correct positioning and strength of membrane binding of the anchor than the acidic residues further upstream. However, when taken together, the results encompass too many discrepancies with the charge zipper idea to render this model likely in the context of precursor processing or E^rns^ membrane binding.

Since the results of the above-described protein analyses did not provide a solid clue for the conservation of the charge distribution pattern in the E^rns^ membrane anchor we tested selected mutants for effects on virus replication. The results of these experiments can be summarized in a way that all mutant genome-like RNAs derived from these constructs were functional replicons. None of the tested RNAs with more than two exchanges allowed the recovery of infectious viruses, which finding strongly supports the functional importance of the charged residues. Single exchange variants could be divided into three different groups. The first type of mutants resulted in viable revertants or pseudorevertants, in which the originally mutated amino acid was replaced by a residue with the same charge. This finding is also true for the double mutant R4E/R9E and underlines the importance of the right charge at the respective position. The second type of mutations (D184R (D4R) and E191R (E1R)) resulted only in functional replicons, while recovery of infectious viruses was not possible. We conducted the transfection experiments several times and passaged the transfected cells repeatedly, but infection experiments of fresh cells with such material always gave negative results. It is not clear, at the moment, why (pseudo)reversion did not occur in these cases, especially since the corresponding versions with alanine exchanges reverted. However, these cases prove that the wrong charge at position 184 or 191 specifically blocks a step in the production of infectious virus particles showing that the E^rns^ membrane anchor is important for this process. The third group of mutants, R206a, D177R, D177a, R199E, and R199a (R6a, D7R, D7a, R9E and R9a, respectively), were able to tolerate the exchange, and exhibited wt growth characteristics. Thus, the correct charge at positions 177 and 199 is not important for virus replication, whereas position 206 can tolerate loss of charge, but not inversion of charge.

An open question concerns the (possible) function of the charged residues in the E^rns^ amphipathic helix on the molecular level. In general, amphipathic helices have been studied more intensely with regard to their hydrophobic side, which is obviously important for interaction with lipids. Besides mere membrane binding, amphipathic helices were found to be involved in deformation of lipid bilayers, recognition of specific lipids or lipid combinations as, e.g., in lipid droplets, sensing, as well as induction of membrane curvature, induction of membrane fission, coating, and protection of lipid structures. For these processes, the nature of the residues on the hydrophobic face and their arrangement is of major importance, but also the sequence on the polar side is crucial, and involvement of charged residues is believed to play a role [53,54,55,56,57]. A much more concrete picture stems from work on positive strand RNA viruses that all use lipid structures for assembly of their RNA replication machinery. Amphipathic helices are of major importance for (sometimes-transient) membrane binding of different viral proteins that organize the assembly of the replication complexes and often rearrangement of cellular membranes into viral replication factories. In these processes, the hydrophilic face of the helices are crucial since they are able to bind other viral and cellular factors, as well as the viral RNA. Examples are the 1a protein of Brome Mosaic virus, nsP1 of Semliki Forest virus, and picornavirus 2C [58,59,60,61,62,63,64,65,66]. A very good example for a virus closely related to pestiviruses is the human hepatitis C virus (HCV), which contains in the amino-terminal region of its NS5A protein an amphipathic helix with an arrangement of positively and negatively charged residue clusters on both sides of a mirror axis. NS5A is an essential component of the HCV replication complex recruiting various other necessary molecules. For this reason, NS5A has to be bound to membranes via its amphipathic helix. It then oligomerizes into a structure providing a so-called “basic railway” able to bind RNA and let it slide. The amino-terminal amphipathic helix is not only responsible for hooking the protein complex to the membrane but is also involved in further functions of NS5A via its hydrophilic face. Mutation analysis has shown that the charged residues found in this helix are important for recruiting NS4B into the replication complex. Exchanges not affecting the membrane binding activity of the helix turned out to be lethal with regard to RNA replication or be compensated by second site reversion in NS4B proving the role of the respective charged residues in the amphipathic helix for protein/protein interaction [59,61,62]. A biochemically similar, but less intensely studied amphipathic helix was also identified at the amino-terminus of BVDV NS5A [63]. It has to be mentioned that the examples presented above all refer to proteins found in the cytoplasm and not inside of cellular compartments or on the surface of virus particles, so that functional roles of the hydrophilic faces of the amphipathic helices cannot be directly compared. However, general lessons to be learned from the well-studied intracellular proteins point to at least two possible roles for the amphipathic helix, apart from anchoring E^rns^ in membranes, namely induction of membrane curvature leading to membrane fission during the budding process and provision of a long and very specific docking area for other molecules. For both of these functions, the charged amino acids would be of major importance. So far, the available data do not allow discrimination between these possible roles. It might turn out that, in fact, both are in part true.

While some of our results obtained with the full-length cDNA constructs are in agreement with the strict conservation of the positioning of the charged residues in the E^rns^ amphipathic helix and clearly demonstrate the importance of the charges presumably in the processes sketched above, the before-mentioned mutations, at positions 177, 199, and 206 are fully tolerated and, thus, the respective positions occupied by charged amino acids seem not to be important for the virus. However, one has to keep in mind that E^rns^ is not only an essential structural protein of pestiviruses, but also an important factor for controlling the host’s innate immune response. The experiments described in our manuscript shed some light on the importance of charged residues in connection with the role of E^rns^ as a structural protein, but do not allow conclusions with regard to its second function. Those charged residues are not important for infectious virus production, but could well be conserved because of a role in repression of innate immune responses that are supposed to be connected with secretion and subsequent internalization of E^rns^ [22]. Again, a certain panel of charged residues could serve here as interaction platform for host factors. This would contribute to the conservation of position, charge, and complementarity of the charges. In this new context, a charge zipper mechanism could still be of crucial functional importance. Further analyses in the natural host are needed to clarify this interesting point.

## 5. Conclusions

The presence and complementary arrangement of charged amino acids located on the hydrophilic face of the amphipathic helix at the carboxy-terminus of pestiviral E^rns^ is conserved among pestivirus species. The respective amino acids have variable influence on the processing of the E^rns^-E1 precursor and the membrane binding/intracellular retention of processed E^rns^ with correct charge and not specific amino acids being important. This variability of the effects of exchanges and the absence of restoring of (nearly) wt features by reciprocal exchanges preserving charge complementarity show that formation of salt bridges between N- and C-terminal parts of the helix via a “charge zipper” is not the key parameter for processing and membrane anchoring. Experiments with virus mutants prove that ~two thirds of the tested charged residues are crucial for recovery of infectious viruses since exchanges show (pseudo)reversion or block release of infectious viruses. The other tested mutants were neutral, indicating that conservation of the respective amino acids is not due to importance for tissue culture replication, but probably plays a role for the virulence factor function of E^rns^. It might be that, for the latter function, a charge zipper is the crucial element, but it is also likely that the hydrophilic face of the helix exposed to the watery surrounding represents an extended area for interaction with other molecules.

## Figures and Tables

**Table 1:** Properties of expression constructs.

**(A)**		
**Name**	**Charge Distribution**	**Exchanges**
wt	− − − − − − − / +++	
1	++++ +++ / +++	E2R/D3R/D8R/E0R/D7R/D4R/E1R
2	++++ − − − / − − −	E2R/D3R/D8R/E0R/R4E/R9E/R6E
3	++ − − +++ / +++	E2R/D3R/D7R/D4R/E1R
4	− − ++ +++ / +++	D8R/E0R/D7R/D4R/E1R
5	++− − − − − / − − −	E2R/D3R/R4E/R9E/R6E
6	− −++ − − − / − − −	D8R/E0R/R4E/R9E/R6E
7	++− − − − − / +++	E2R/D3R
8	− − ++ − − − / +++	D8R/E0R
9	++++ − − − / +++	E2R/D3R/D8R/E0R
10	− − − − +++ / +++	D7R/D4R/E1R
11	− − − − − − − / − − −	R4E/R9E/R6E
**(B)**		
**Name**	**Charge Distribution**	**Exchanges**
wt	− − − / + + +	
10	+ + + / + + +	D7R/D4R/E1R
11	− − − / − − −	R4E/R9E/R6E
12	+ + − / + − +	D7R/D4R/R9E
13	+ + − / + + −	D7R/D4R/R6E
14	+ + − / + − −	D7R/D4R/R9E/R6E
15	+ − + / − + −	D7R/E1R/R4E/R6E
16	+ + + / + − −	D7R/D4R/E1R/R9E/R6E
17	+ + − / − − −	D7R/D4R/R4E/R9E/R6E
18	+ + + / − − −	D7R/D4R/E1R/R4E/R9E/R6E
19	+ + − / + + +	D7R/D4R
20	+ − + / + + +	D7R/E1R
21	− + − / − + +	D4R/R4E
22	− − + / − + +	E1R/R4E
23	+ − − / + + −	D7R/R6E
24	− − + / + − +	E1R/R9E
25	− − + / + + −	E1R/R6E
26	− − − / + − +	R9E/R6E
27	− − − / − − +	R4E/R9E
28	− − − / − + −	R4E/R6E
29	+ − − / + + +	D7R
30	− + − / + + +	D4R
31	− − + / + + +	E1R
32	− − − / − + +	R4E
33	− − − / + − +	R9E
34	− − − / + + −	R6E
**(C)**		
**Name**	**Charge Distribution**	**Exchanges**
wt	− − − / + + +	
35	− − a / + + +	E1a
36	− − − / a + +	R4a
37	a − a / + + +	D7a/E1a
38	a − − / a + +	D7a/R4a
39	− a − / a + +	D4a/R4a
40	− − a / a + +	E1a/R4a
41	− − a / + a +	E1a/R9a
42	− − − / a a +	R4a/R9a
43	− − − / a + a	R4a /R6a
44	− a a / a + +	D4a/E1a/R4a
45	− − a / a a +	E1a/R4a/R9a
46	a a a / + + +	D7a/ D4a/E1a
47	− − − / a a a	R4a/R9a/R6a
48	− − − / − + +	R4D
49	− − − / + + +	R4K
50	a − − / + + +	D7a
51	− a − / + + +	D4a
52	− − − / + a +	R9a
53	− − − / + + a	R6a

**Table 2:** Summary of the results of experiments conducted with the infectious CSFV cDNA clone. Column 1 lists the number of the individual mutant (same as in Table 1 and the figures). Column 2: amino acid exchanges encoded by the construct; column 3: charge distribution (“a” represents exchange for alanine); column 4: determined E^rns^-E1 processing efficiency; column 5; determined E^rns^ secretion level; column 6: functionality as replicon; column 7: recovery of infectious virus, yes or no; column 8: sequencing results after isolation of RNA from third passage cells and RT-PCR amplification of CSFV sequence, numbering code as in column 2 and Figure 1; column 9: charge distribution pattern in E^rns^ of the recovered virus. N.t. = not tested; n.a. = not applicable. Yellow backcolor: no virus recovered, Blue backcolor: recovered viruses represent revertants or pseudorevertants.

Construct	Mutation	Charge	Precursor	Secretion	Replicon	Inf. Virus	Seq	Charge Rec. Virus
wt	-	− − −/+ + +	14%	7%	yes	yes	wt	− − −/+ + +
ΔE^rns^	deletion of E^rns^ gene	n.a.	n.a.	n.a.	yes	no	n.a.	n.a.
12	D7R/D4R/R9E	+ + −/+ − +	19%	29%	yes	no	n.a.	n.a.
18	D7R/D4R/E1R/R4E/R9E/R6E	+ + +/− − −	30%	43%	yes	no	n.a.	n.a.
27	R4E/R9E	− − −/− −+	44%	63%	yes	yes	4K/9R	− − −/+ + +
29	D7R	+ − −/+ + +	24%	5%	yes	yes	7R	+ − −/+ + +
30	D4R	− + −/+ + +	23%	26%	yes	no	n.a.	n.a.
31	E1R	− − +/+ + +	16%	28%	yes	no	n.a.	n.a.
32	R4E	− − −/− + +	41%	26%	yes	yes	4K or 4R	− − −/+ + +
33	R9E	− − −/+ − +	21%	29%	yes	yes	9E	− − −/+ − +
34	R6E	− − −/+ + −	22%	28%	yes	yes	6K	− − −/+ + −
								
50	D7a	a − −/+ + +	14%	n.t.	yes	yes	7a	a − −/+ + +
51	D4a	− a −/+ + +	16%	n.t.	yes	yes	4D	− − −/+ + +
35	E1a	− − a/+ + +	12%	n.t.	yes	yes	1E	− − −/+ + +
36	R4a	− − −/a + +	19%	n.t.	yes	yes	4R	− − −/+ + +
52	R9a	− − −/+ a +	16%	n.t.	yes	yes	9a	− − −/+ a +
53	R6a	− − −/+ + a	n.t.	n.t.	yes	yes	6a	− − −/+ + a

## Data Availability

Data is contained within the article, no additional data used.
